# Whole-Tumor Histogram and Texture Imaging Features on Magnetic Resonance Imaging Combined With Epstein-Barr Virus Status to Predict Disease Progression in Patients With Nasopharyngeal Carcinoma

**DOI:** 10.3389/fonc.2021.610804

**Published:** 2021-03-09

**Authors:** Qiao Li, TingTing Wang, Yan Huang, Qin Li, PeiYao Liu, Robert Grimm, CaiXia Fu, YunYan Zhang, Yajia Gu

**Affiliations:** ^1^Department of Radiology, Fudan University Shanghai Cancer Center, Shanghai, China; ^2^Department of Oncology, Shanghai Medical College, Fudan University, Shanghai, China; ^3^Department of Radiation Oncology, Fudan University Shanghai Cancer Center, Fudan University, Shanghai, China; ^4^Magnetic Resonance Application Predevelopment, Siemens Healthcare, Erlangen, Germany; ^5^Magnetic Resonance Applications Development, Siemens Shenzhen Magnetic Resonance Ltd., Shenzhen, China; ^6^Department of Radiology, Shanghai Proton and Heavy Ion Center, Shanghai, China

**Keywords:** nasopharyngeal carcinoma, Epstein-Barr virus, histogram, texture feature, disease progression

## Abstract

**Purpose:** We aimed to investigate whether Epstein–Barr virus (EBV) could produce differences on MRI by examining the histogram and texture imaging features. We also sought to determine the predictive value of pretreatment MRI texture analyses incorporating with EBV status for disease progression (PD) in patients with primary nasopharyngeal carcinoma (NPC).

**Materials and Methods:** Eighty-one patients with primary T2-T4 NPC and known EBV status who underwent contrast-enhanced MRI were included in this retrospective study. Whole-tumor-based histogram and texture features were extracted from pretreatment T1-weighted imaging (T1WI), T2-weighted imaging (T2WI), and contrast-enhanced (CE)-T1WI images. Mann–Whitney *U*-tests were performed to identify the differences in histogram and texture parameters between EBV DNA-positive and EBV DNA-negative NPC images. The effects of clinical variables as well as histogram and texture features were estimated by using univariate and multivariate logistic regression analyses. Receiver operating characteristic (ROC) curve analysis was used to predict the EBV status and PD. Finally, an integrated model with the best performance was built.

**Results:** Of the 81 patients included, 54 had EBV DNA-positive NPC, and 27 had EBV DNA-negative NPC. Patients who were tested EBV DNA-positive had higher overall stage (*P* = 0.016), more lymphatic metastases (*p* < 0.0001), and easier distant metastases (*P* = 0.026) than the patients who were tested EBV DNA-negative. Tumor volume, T1WI_*Skewness*_ and T2WI_*Kurtosis*_ showed significant differences between the two groups. The combination of the three features achieved an AUC of 0.783 [95% confidence interval (CI) 0.678–0.888] with a sensitivity and specificity of 70.4 and 74.1%, respectively, in differentiating EBV DNA-positive tumors from EBV DNA-negative tumors. The combination of overall stage and tumor volume of T2WI_*Kurtosis*_ and EBV status was the most effective model for predicting PD in patients with primary NPC. The overall accuracy was 84.6%, with a sensitivity and specificity of 93.8 and 66.2%, respectively (AUC, 0.800; 95% CI 0.700–0.900).

**Conclusion:** This study demonstrates that MRI-based radiological features and EBV status can be used as an aid tool for the evaluation of PD, in order to develop tailored treatment targeting specific characteristics of individual patients.

## Introduction

Nasopharyngeal carcinoma (NPC) is an epithelial carcinoma arising from the nasopharyngeal mucosal lining with distinct geographic distributions and is endemic in Southern China and Southeast Asia. Epstein–Barr virus (EBV) has been linked to the development of lymphoid and epithelial cell cancers, with a predominance of NPC. In the latest Union for International Cancer Control/American Joint Committee on Cancer (UICC/AJCC) TNM (tumor-node-metastasis) staging system, EBV DNA has been established as robust evidence for the presence of early-stage NPC (1). Establishing EBV status in patients with NPC is clinically significant because the treatments and prognoses are different between patients who were EBV DNA-positive and patients who were EBV DNA-negative. Plasma EBV DNA is considered a promising marker for tumor diagnoses, disease monitoring, and prognosis predictions in patients with NPC. Therefore, this test is widely used in clinical practice (2, 3).

MRI is a traditional and important tool for pretreatment staging and therapeutic strategy development for patients with NPC. Moreover, functional MRI includes diffusion-weighted imaging (DWI) (4) and perfusion-weighted imaging (PWI) (5), which have proved useful in the evaluation of tissue properties and tumor behaviors. However, traditional MRI is mainly based on the whole-tumor presentations and does not consider intratumoral heterogeneity. Radiomics, a prospective technique, can comprehensively analyze tumor phenotypes by converting medical images into minable data and extracting abundant quantitative features as imaging biomarkers. Evidence from previous studies showed that radiomic features could be helpful for the exact segmentation of gross tumor volume (6), personalized risk stratifications (7), and individual treatment decisions (8).They could serve as prognostic factors in patients with NPC (9, 10).

Limited data have distinguished the imaging features of primary EBV DNA-positive NPC from EBV DNA-negative NPC. Prior data describing these differences focused on primarily delineating the extent of lesions and displaying lymph node metastases. For example, a significant correlation was noted between pretreatment EBV DNA levels and disease stages (11). Plasma EBV-DNA is a critical molecular NPC biomarker, and imaging histogram and texture analyses could provide adequate details about NPC tumors. Therefore, combining EBV DNA levels with MRI histogram and texture features could improve NPC prognosis predictions.

In this study, we investigated the potential of MRI histogram and texture features, extracted from multiple modalities, to distinguish patients with EBV DNA-positive NPC from patients with EBV DNA-negative NPC. We also investigated the predictive value of pretreatment MRI texture analyses in combining clinical features and EBV status to determine disease progression (PD) in patients with primary NPC.

## Materials and Methods

### Patients

This retrospective single-center study was approved by our institutional review board (IRB). One hundred and sixty-three patients, who had undergone radiation therapy of NPC at the Shanghai Cancer Center, Fudan University, from January 2018 to March 2019, were reviewed. The inclusion criteria were patients with (a) biopsy-proven primary NPC; (b) stage II–IV disease according to the eighth edition of the UICC/AJCC TNM staging system; (c) absence of secondary malignancies, pregnancies, or lactations; (d) MRI scans available for review, including pretreatment T1-weighted imaging (T1WI), T2-weighted imaging (T2WI), and contrast-enhanced (CE)-T1WI; (e) an absence of treatments, such as surgery, radiotherapy (RT), or chemoradiotherapy before the MRI scans; (f) PET examinations performed to evaluate metastatic sites before treatment; (g) blood samples obtained at baseline for the enumeration of EBV DNA copy numbers; and (h) completed clinical follow-up information. The histological subtype of the patients' tumors was categorized according to WHO standards and included type I (differentiated keratinizing carcinoma), type II (differentiated non-keratinizing carcinoma), and type III (undifferentiated non-keratinizing carcinoma). Finally, a total of 81 patients with primary NPC, who met the criteria, were identified.

Baseline clinical variables were collected, including age, gender, T stage, N stage, histology, and immunoglobulin A antibody testing against EBV capsid antigen (VCA-IgA) or early antigen (EA-IgA). The primary endpoint of this study was progression-free survival (PFS), which was defined as the time from the start of the MRI examinations until the date of local or distant PD.

### Radiation Therapy and Follow-Up Visits

All patients received intensity-modulated radiation therapy (IMRT) or three-dimensional conformal RT to treat the primary tumors and cervical adenopathies. Total radiation doses ranged from 66 to 70.4 grays (Gy). Neoadjuvant or adjuvant chemotherapy and/or concurrent chemotherapy with RT were also performed, according to the National Comprehensive Cancer Network clinical practice guidelines for NPC. Patient follow-up visits occurred every 3 months. The minimum follow-up time for patients without local recurrence was 20 months after the first MRI examination. At each follow-up visit, medical histories were taken and physical examinations, MRI of nasopharyngeal-neck, thoracic CT scans, abdominal sonography, and whole-body bone scintigraphy were performed. Furthermore, PET-CT scans were arranged, if needed. All follow-ups ended in December 2020.

### Plasma EBV DNA Assay

DNA was extracted from plasma using the Quantitative Diagnostic Kit for EBV-DNA (Daan Gene, Zhongshan University, China). The plasma EBV DNA concentrations in patients were measured with a quantitative (qPCR) assay before treatment. Amplifications were carried out using an ABI QuantStudio™ Dx Real-Time PCR Instrument (Thermo Fisher Scientific, Waltham, Massachusetts, USA). At our institution, the plasma EBV DNA was considered undetectable at concentrations <500 copies/ml. Patients who had more than 500 copies/ml were placed into the EBV-positive group.

### MRI

MRI was performed using a 3T scanner (MAGNETOM Skyra, Siemens Healthcare, Erlangen, Germany) with a dedicated 16-channel head/neck coil. The MRI protocol included, axial T1-weighted turbo scan echo (TSE) (TR, 500 ms; TE, 6.5 ms; slice spacing, 5.4 mm, voxel size, 0.73 × 0.71 × 4.5 mm^3^, matrix, 320 × 372, field of view (FOV), 270 mm, FOV phase 84.4%), T2-weighted TSE (TR, 2,500 ms; TE, 78 ms; voxel size, 0.70 × 0.70 × 4.5 mm^3^, slice spacing, 5.4 mm, matrix, 384 × 324, FOV, 270 mm, FOV phase 84.4%), contrast-enhanced fat-saturated T1-weighted 3D fast low-angle shot (FLASH) with an resolution of 0.7 × 0.7 × 3 mm^3^. A rapid bolus of gadolinium contrast agent (Magnevist, Bayer HealthCare Pharmaceuticals Inc., Wayne, USA) was injected intravenously at a dose of 0.1 mmol/kg body weight.

### Image Analysis

Two radiologists with 7 (LQ) and 8 years (WTT) experience in diagnostic MRI assessed all of the images for each patient and staged the tumors by consensus according to the established staging system. All manual segmentations of the tumor were performed by these two radiologists in a blinded fashion. Prototypic MR Multiparametric Analysis software (Siemens Healthcare, Erlangen, Germany) was used to perform histogram and texture analyses by the radiologists. The processing workflow included the following five steps:

- Data loading. MR data (T1WI, T2WI, and CE-T1WI) were loaded onto the software.
- Image registration. Automated registration was performed for the input MRI data using rigid plus non-rigid registration.
- ROI drawing. For CE-T1WI analysis, foreground and background seed points were manually drawn inside and outside the tumor, respectively, on the three multiplanar reconstruction (MPR) planes.
- Segmentation. The segmentation of whole tumor was executed based on these seed points with a random-walker algorithm. Manual adjustments for the segmentations were performed, if necessary. Then, the segmented regions of interest (ROIs) were automatically copied to the T1WI and T2WI data.
- Histogram and texture analyses. Whole-tumor-based volume size and histogram parameters, including the mean, median, SD, fifth percentile, 95th percentile, skewness, and kurtosis, were extracted from the input images. Texture parameters, including difference entropy, difference variance, contrast, and entropy were also extracted.

**Figure 1:**
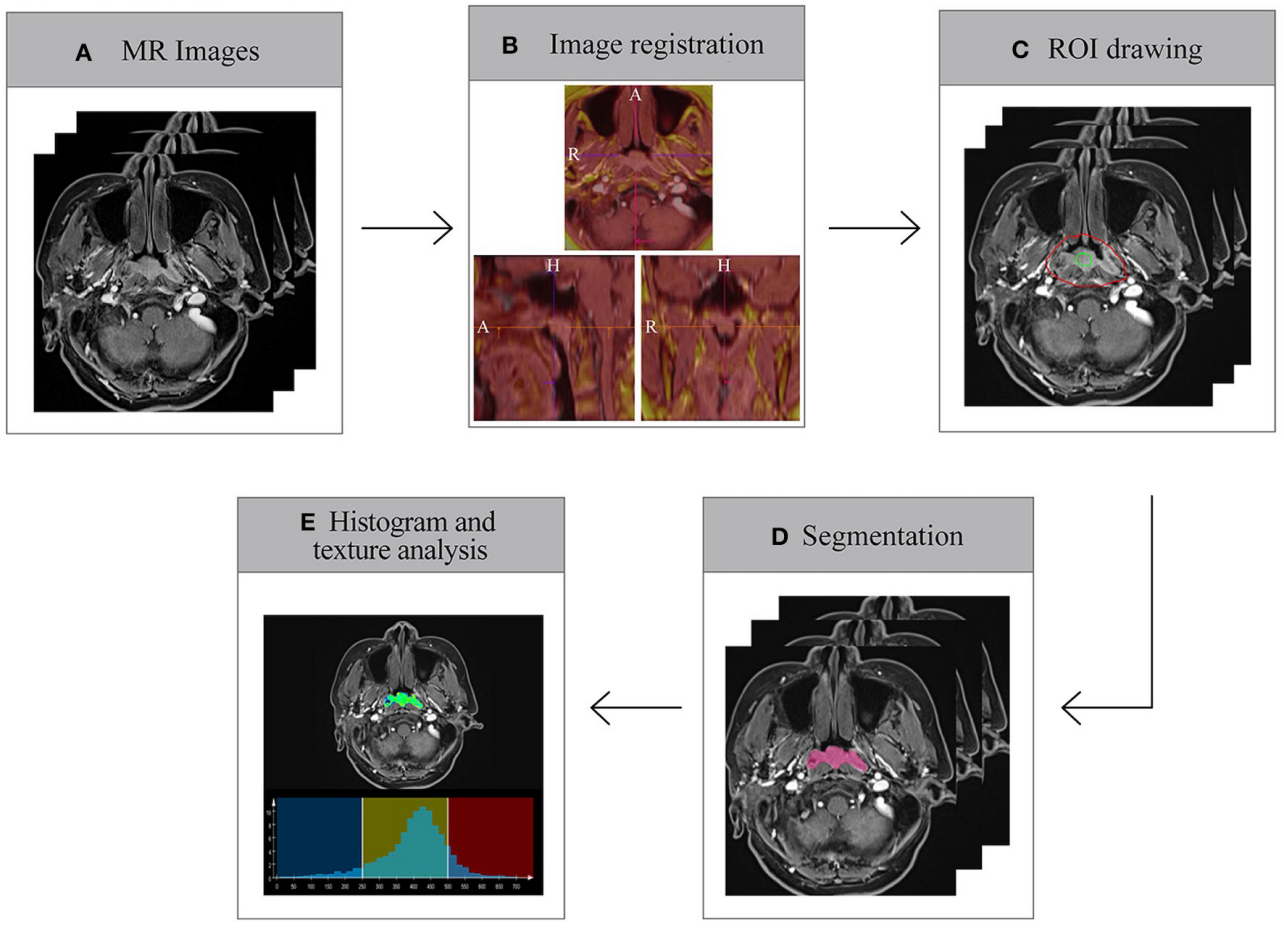
Schematic diagram of data processing.

The steps of the imaging analysis are illustrated in Figure 1.

### Statistical Analyses

Categorical variables were summarized as percentages and compared using Pearson's chi-square test or Fisher's exact test, when appropriate. The intraobserver agreement was calculated based on the measurements of the tumor volume done by two radiologists using an interclass correlation coefficient (ICC). For continuous variables, the independent-sample *t-*test or Mann–Whitney *U*-test was used to compare the differences between the two groups. The differences among the three groups were compared with Kruskal–Wallis 1-way ANOVA followed by a Bonferroni correction. The abilities of each independent predictor or combined predictors in predicting the EBV status were assessed using ROC analyses. The area under the ROC curve (AUC), sensitivity, specificity, and accuracy of each model were calculated. The effects of clinical variables as well as the histogram and texture features were estimated by the univariate logistic regression analysis. The predictive factors with value of *p* < 0.1 in univariate logistic regression analysis were chosen for the multivariate logistic regression. ROC curve analyses were used to build an integrated model with best performance to predict PD. All analyses were performed using SPSS (version 22; IBM, Armonk, NY, USA). Statistical significance was determined with a value of *p* < 0.05.

## Results

### The Intraobserver Consistency Analysis

Interobserver agreement between the two radiologists was excellent [ICC = 0.921, 95% confidence interval (CI), 0.880–0.949, *p* < 0.0001].

### Clinical Characteristics

Baseline clinicopathologic characteristics of 81 patients with NPC are summarized in Table 1. A total of 27 patients were EBV DNA-negative (33.3%), and 54 patients were EBV DNA-positive (66.7%). Patients who were EBV DNA-positive had more lymphatic metastases compared with patients who were EBV DNA-negative (*p* < 0.05) and were more likely to present with distant metastases (*p* < 0.05). The overall stage also showed significant differences between the two groups (*p* < 0.05), and patients with stage IV NPC were present in higher proportions in the EBV DNA-positive group compared with those in the EBV DNA-negative group. Significantly, higher VCA-IgA titers were observed in patients with EBV DNA-positive NPC (*p* < 0.05) compared with patients with EBV-negative NPC. Age, sex, smoking, and drinking histories, T stages, and EA-IgA titers were not different between the two groups. The PFS of the EBV DNA-negative group was slightly longer than the EBV DNA-positive group; however, no significant differences were seen.

**Table 1:** Baseline characteristics of patients with nasopharyngeal carcinoma (NPC).

**Characteristics**	**All** **(*n* = 81)**	**EBV DNA-negative** **(*n* = 27)**	**EBV DNA-positive** **(*n* = 54)**	***P*-value**
Age (years)	49.3 ± 11.8	50.9 ± 12.0	48.5 ± 11.7	0.913
Sex				
Male	62 (76.5%)	21 (77.8%)	41 (75.9%)	0.853
Female	19 (23.5%)	6 (22.2%)	13 (24.1%)	
Smoking (*n*, %)	34 (42.0%)	9 (42.9%)	25 (46.3%)	0.265
Drinking (*n*, %)	18 (22.2%)	7 (25.9%)	11 (20.4%)	0.571
Pathologic type (*n*, %)				
WHO type I	0 (0%)	0 (0%)	0 (0%)	1.000
WHO type II	7 (8.6%)	2 (7.4%)	5 (9.3%)	
WHO type III	74 (91.4%)	25 (92.6%)	49 (90.7%)	
T stage (*n*, %)				
2	31 (38.3%)	10 (37.0%)	21 (38.9%)	0.869
3	26 (32.1%)	8 (29.6%)	18 (33.3%)	
4	24 (29.6%)	9 (33.4%)	15 (27.8%)	
N stage (*n*, %)				
0	8 (9.9%)	6 (22.2%)	2 (3.7%)	0.000
1	19 (23.4%)	14 (51.9%)	5 (9.3%)	
2	28 (34.6%)	5 (18.5%)	23 (42.6%)	
3	26 (32.1%)	2 (7.4%)	24 (44.4%)	
M stage (*n*, %)				
0	71 (87.7%)	27 (100%)	44 (81.5%)	0.026
1	10 (12.3%)	0 (0%)	10 (16.5%)	
Overall stage (*n*, %)				
II	12 (14.8%)	8 (29.6%)	4 (7.4%)	0.016
III	25 (30.9%)	9 (33.4%)	16 (29.6%)	
IV	44 (54.3%)	10 (37.0%)	34 (63.0%)	
Anti-EBV capsid antigen IgA antibody (VCA-IgA)	7.5 (4.7–14.4)	5.9 (3.9–10.1)	8.0 (4.4–16.9)	0.03
Early antigen IgA antibody (EA-IgA)	4.8 (1.5–11.9)	1.7 (0.9–9.3)	5.2 (2.0–12.9)	0.072
Progression-free survival (months)	23.0 (20.5–26.5)	23.0 (21.0–26.5)	22.8 (20.4–26.5)	0.329

*T, tumor; N; nodal; M, metastasis*.

### Histogram and Texture Analyses

**Figure 2:**
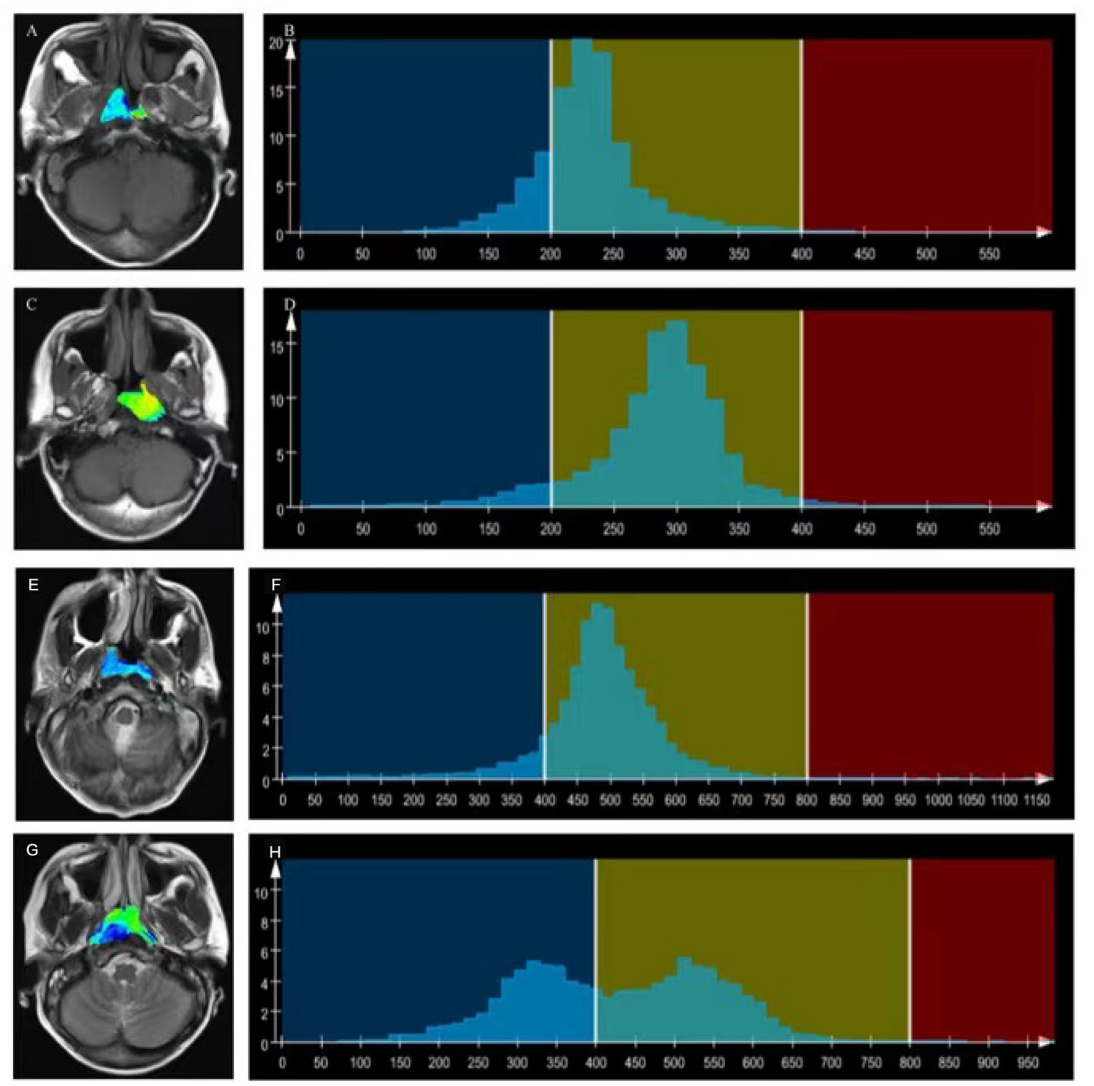
T1WI overlaid with color maps **(A,C)** and histograms of whole-tumor using T1WI maps **(B,D)**. A T1WI histogram from **(A)** male patient with EBV DNA NPC showed higher T1WI_*Skewness*_ (T1WI_*Skewness*_ = 0.737). **(B)** than that of a male patient with EBV DNA-positive NPC (T1WI_*Skewness*_ = 0.435). **(D)** T2WI) overlaid with color maps **(E,G)** and histograms of whole-tumor using T2WI maps **(F,H)**. A T2WI histogram from a female patient with EBV DNA-negative NPC showed obviously higher T2WI_*Kurtosis*_ (T2WI_*Kurtosis*_ = 9.004) **(F)** than that of a male patient with EBV DNA-positive NPC (T2WI_*Skewness*_ = −0.958) **(H)**. T1WI, T1-weighted imaging; EBV, Epstein–Barr virus; NPC, nasopharyngeal carcinoma, T2WI, T2-weighted imaging.

**Figure 3:**
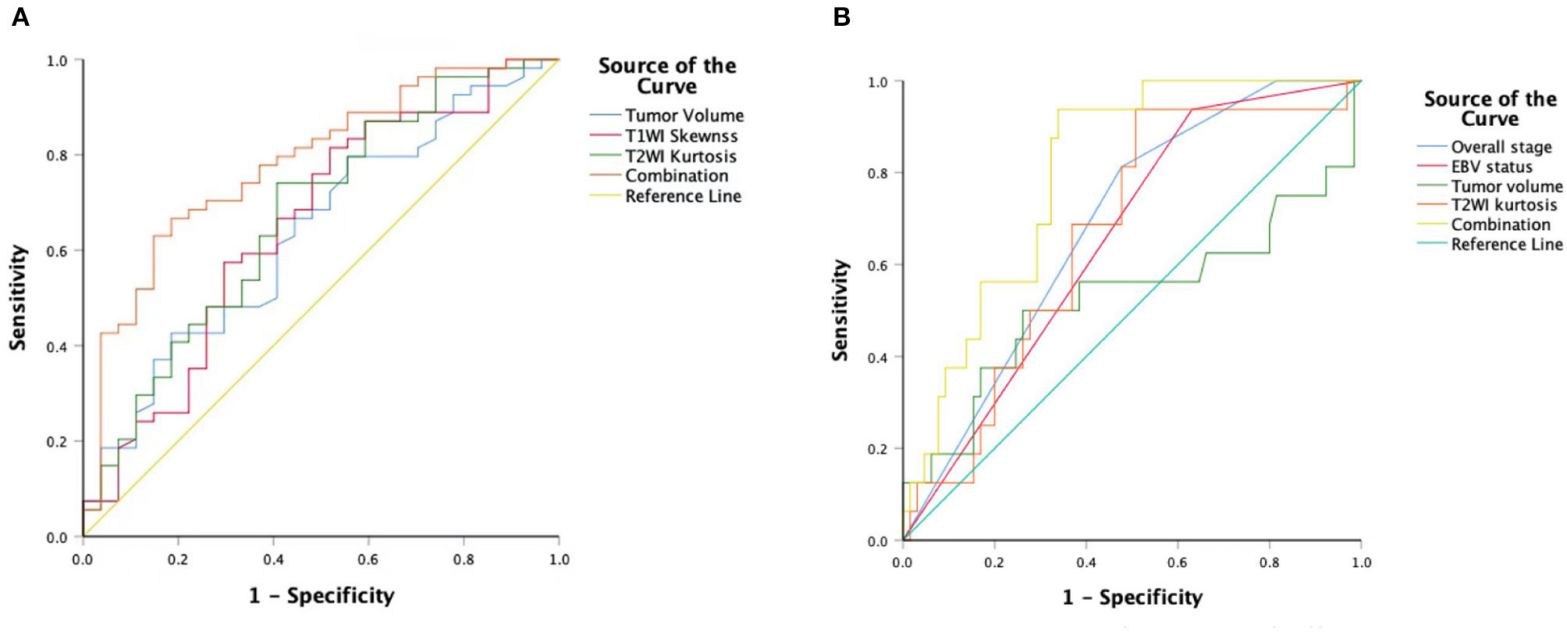
An ROC curve to differentiate the EBV DNA-negative group compared with the EBVDNA-positive group using T1WI_*Skewness*_, T2WI_*Kurtosis*_, tumor volumes, and a combination of the three variables **(A)**. An ROC curve to differentiate the PD from the non-PD group using overall stages, tumor volumes, T2WI_*Kurtosis*_, and EBV statuses and a combination of the four variables **(B)**. ROC, receiver operating characteristic; T1WI, T1-weighted imaging; EBV, Epstein–Barr virus; T2WI, T2-weighted imaging; PD, disease progression.

T1WI_*Skewness*_, T2WI_*Kurtosis*_, and tumor volume between the EBV DNA-positive and EBV DNA-negative groups were statistically different. T1WI_*Skewness*_ and T2WI_*Kurtosis*_ were significantly higher in the EBV DNA-negative group than those in the EBV DNA-positive group [153.2 (37.9–227.0) vs. 66.3 (16.9–153.7), *p* < 0.05; 200.8 (101.8–425.1) vs. 119.6 (33.7–211.0), *p* < 0.05, respectively] (Figure 2). In addition, tumor volume was significantly lower in the EBV DNA-negative group [6.4 (3.9–10.4) vs. 8.5 (5.7–15.4), *p* < 0.05]. The univariate analyses of extracted features are shown in Tables 2, 3. When the sensitivity and specificity of T1WI_*Skewness*_, T2WI_*Kurtosi*_, and the NPC tumor volume were compared using the ROC analysis, the AUCs were 0.653 vs. 0.671 vs. 0.636. Multivariable logistic regression analyses of combined tumor volume, T1WI_*Skewness*_, and T2WI_*Kurtosis*_ values resulted in a model that allowed the correct classification in 71.6% of the cases, which corresponded to an AUC of the ROC curve of 0.783 (sensitivity, 70.4%; specificity, 74.1%). The accuracy was also higher than any single predictor mentioned above. The performance of these variables in differentiating the two groups is shown in Table 4 and Figure 3A.

**Table 2:** Univariate analyses of histogram features for differentiating Epstein-Barr virus (EBV) DNA-negative from EBV DNA-positive patients with nasopharyngeal carcinoma.

**Variable**	**EBV DNA-negative (*n* = 27)**	**EBV DNA-positive (*n* = 54)**	***P*-value**
Tumor volume (cm^3^)	6.4 (3.9–10.4)	8.5 (5.7–15.4)	**0.047**
T1WI-based parameters			
Mean	280.4 (252.7–299.7)	285.9 (259.7–313.4)	0.373
SD	57.1 (41.7–85.9)	49.4 (40.4–60.5)	0.148
Median	295.5 (251.5–295.5)	280.5 (260.0–312.8)	0.209
5%	193.0 (174.5–225.5)	212.5 (187.5–229.5)	0.060
95%	381.5 (328.5–477.5)	360.0 (333.5–403.3)	0.534
Skewness*10^−2^	153.2 (37.9–227.0)	66.3 (16.9–153.7)	**0.026**
Kurtosis*10^−2^	451.0 (220.9–1395.9)	341.3 (159.2–694.8)	0.098
T2WI-based parameters			
Mean	488.1 (445.6–523.9)	501.8 (452.2–541.4)	0.241
SD	109.4 (96.4–127.1)	114.1 (96.2–134.4)	0.589
Median	485.5 (459.5–524.5)	507.0 (458.8–546.8)	0.293
5%	282.5 (254.5–333.5)	313.0 (263.3–353.5)	0.239
95%	669.5 (612.5–709.0)	683.5 (629.0–722.5)	0.471
Skewness*10^−2^	37.8 (−1.9–71.4)	24.7 (−22.9–63.6)	0.266
Kurtosis*10^−2^	200.8 (101.8–425.1)	119.6 (33.7–211.0)	**0.012**
CE-T1WI-based parameters			
Mean	561.3 (507.7–608.8)	572.9 (524.7–627.9)	0.321
SD	102.9 (82.2–118.6)	105.7 (87.4–119.3)	0.406
Median	565.5 (495.5–627.5)	576.0 (527.3–638.3)	0.417
5%	371.5 (335.5–412.5)	398.0 (336.8–448.5)	0.133
95%	711.5 (615.5–798.5)	733.0 (674.5–799.0)	0.408
Skewness*10^−2^	−34.8 [−56.8–(−5.9)]	−21.2 [−53.4–(−4.6)]	0.314
Kurtosis*10^−2^	66.8 (24.3–132.0)	63.9 (26.1–126.1)	0.775

*If p < 0.05, it will be highlighted in bold type, which means statistical significance*.

**Table 3:** Univariate analyses of texture features for differentiating patients with EBV DNA-negative NPC from patients with EBVDNA-positive NPC.

**Variable**	**EBV DNA-negative (*n* = 27)**	**EBV DNA-positive (*n* =5 4)**	***P*-value**
T1WI-based texture parameters			
Difference entropy*10^−2^	79.2 (71.1–95.0)	75.0 (66.8–89.6)	0.367
Difference variance*10^−2^	20.0 (27.4–16.4)	18.4 (14.8–23.7)	0.279
Contrast*10^−2^	43.2 (33.3–75.5)	39.8 (28.8–57.0)	0.201
Entropy*10^−2^	123.39 (105.3–147.9)	118.5 (92.8–142.7)	0.316
T2WI-based parameters			
Difference entropy*10^−2^	118.9 (106.2–127.6)	117.9 (109.3–129.0)	0.976
Difference variance*10^−2^	44.0 (37.9–53.1)	44.1 (36.1–52.5)	0.814
Contrast*10^−2^	140.8 (105.9–175.5)	127.0 (105.9–171.1)	0.589
Entropy*10^−2^	207.5 (175.2–225.4)	203.8 (188.1–219.8)	0.745
CE-T1WI-based parameters			
Difference entropy*10^−2^	161.3 (145.9–173.5)	164.9 (152.2–177.5)	0.304
Difference variance*10^−2^	96.9 (73.1–126.9)	99.9 (76.4–139.0)	0.783
Contrast*10^−2^	338.8 (239.4–455.9)	353.1 (258.6–483.2)	0.452
Entropy*10^−2^	265.8 (248.9–289.0)	278.1 (259.4–294.9)	0.381

**Table 4:** The diagnostic performance MRI histogram and texture features to differentiate patients with EBV DNA-negative NPC from patients with EBV DNA-positive NPC.

**Variable**	**Sensitivity**	**Specificity**	**Accuracy**	**AUC**	**95% CI**
Tumor volume	0.667	0.519	0.618	0.636	0.509–0.763
T1WI*_*Skewness*_*	0.593	0.667	0.618	0.653	0.521–0.784
T2WI*_*Kurtosis*_*	0.741	0.593	0.692	0.671	0.544–0.799
Combination	0.704	0.741	0.716	0.783	0.678–0.888

*AUC, area under the curve; CI, confidence interval*.

When comparing the different tumor stage groups (T2 vs. T3 vs. T4), tumor volume sizes increased from T2 to T4 (*p* < 0.05). The T1WI-based histogram and texture features, including the mean, median, 95th percentile, contrast, and entropy, showed significant differences among the three groups. In particular, seven T1WI-based histogram and texture features of the T2 group were significantly higher than those of the T3 group, while eight T1WI-based histogram and texture features of the T2 group were significantly higher than those of the T4 group. However, no significant differences were found between the T3 and T4 groups based on the histogram and texture features. Histogram and texture features extracted from T2WI, including the mean, median, skewness, and difference variance, showed significant differences among the three groups. Only one T2WI-based histogram and texture feature showed a significant difference between the T2 and T3 groups, and between the T3 and T4 groups. Moreover, four features of the T2 group were significantly different from those of the T4 group. However, none of the CE-T1WI-based histogram and texture features revealed significant differences among the three groups. CE-T1WI_*DiffVariance*_ and CE-T1WI_5%_ were higher in the T2 group compared with the T4 group. Histogram and texture parameter values of the T stages are summarized in Supplementary Table 1.

### Disease Progression

Sixteen patients showed PD with a PFS mean of 12.5 months (range, 6.9–19.4 months). Among these patients, seven had local-regional recurrences, five had distant metastases, and four had distant relapses. The patients with local-regional recurrences, who showed progressive cervical adenopathy on MRI or neoplasm on nasopharyngoscopy, were subsequently confirmed by fine-needle aspiration biopsy. The distant metastases or relapses were confirmed with PET-CT. Sixty-five patients were included in the non-PD group with a mean PFS of 24.0 months (range, 21.5–26.5 months).

Univariate logistic regression analysis revealed that M stage, overall stage, tumor volume, T2WI_*Kurtosis*_, and EBV status were significantly associated with PD. Since the M stage was reflected in the overall stage, only the overall stage was included in further statistical analyses. Finally, the overall stage, tumor volume, T2WI_*Kurtosis*_, and EBV status were entered into the multivariable logistic regression analyses. The performance of these variables in differentiating PD group from non-PD group is shown in Table 5. The final regression model achieved an accuracy of 84.6% (sensitivity 93.8%, specificity 66.2%, AUC 0.800, and 95% CI 0.700–0.900). The performance of clinical and imaging features in differentiating the patients with PD from patients with non-PD of NPC is shown in Figure 3B.

**Table 5:** The diagnostic performance of clinicoradiological features to differentiate disease progression (PD) from patients with non-PD NPC.

**Variable**	**PD (*n* = 16)**	**non-PD (*n* = 65)**	***P*-value**	**OR (95% CI)**
Age	47.56 ± 14.04	49.68 ± 11.22	0.518	/
Sex (*n*, %)				
Male	15 (93.8%)	47 (72.3%)	0.102	/
Female	1 (6.3%)	18 (27.7%)		
Smoking (*n*, %)	8 (50%)	26 (40%)	0.469	/
Drinking (*n*, %)	2 (12.5%)	16 (24.6)	0.307	/
T stage (*n*, %)				
2	4 (25%)	27 (41.5%)	0.254	/
3	6 (37.5%)	20 (30.8%)		
4	6 (37.5%)	18 (27.7%)		
N stage (*n*, %)				
0	1 (6.3%)	7 (10.8%)	0.175	/
1	2 (12.5%)	17 (26.2%)		
2	6 (37.5%)	22 (33.8%)		
3	7 (43.8%)	19 (29.2)		
M stage (*n*, %)				
0	10 (62.5%)	60 (92.3%)	**0.005**	7.200 (1.843–28.126)
1	6 (37.5%)	5 (7.7%)		
Overall stage (*n*, %)				
II	0 (0%)	12 (18.5%)	**0.021**	4.091 (1.241–13.489)
III	3 (18.7%)	22 (33.8%)		
IV	13 (81.7%)	31 (47.7%)		
Tumor volume	10.85 (1.7–90.6)	7.8 (1.6–33.7)	**0.097**	1.039 (0.993–1.088)
T1WI*_*Skewness*_*	0.664 (0.434–1.792)	0.816 (0.191–1.758)	0.539	/
T2WI*_*Kurtosis*_*	0.97 (−0.34–6.43)	1.49 (−0.96–9.04)	0.090	0.692 (0.452–1.059)
EBV status (n, %)	39 (60.0)	15 (93.8)	**0.041**	8.780 (1.090–70.705)

*OR, odds ratio; AUC, area under the curve; CI, confidence interval; N, nodal; M, metastasis. If p < 0.05, it will be highlighted in bold type, which means statistical significance*.

## Discussion

Using histogram and texture feature analysis, we found that MRI imaging features were closely in association with the EBV status and PD in patients with NPC. In this study, we built a predictive model that combined the imaging features and clinical variables to evaluate the risk of PD in patients with NPC before the initial treatment. The predictive model provides a visual tool for optimal clinical decisions, enabling clinicians to perform inexpensive and earlier identification of patients with NPC, who have a high risk of PD.

Plasma EBV DNA concentrations are believed to be associated with tumor burden (12, 13). Patients with positive EBV DNA were characterized as having more advanced T and N stages (14), which is in accordance with our research results. Although no differences between the EBV DNA-positive and EBV DNA-negative groups were seen for T stages, there were a higher number of EBV-positive patients with stage IV NPC. In this study, T1 cases were excluded because these cases had segmentation and assessment issues. However, a greater number of patients who were tested EBV DNA-negative had early-stage disease compared with patients who were tested EBV DNA-positive (15). We also found that tumor volume was another reliable choice for the radiologic evaluations of tumor burden because patients with positive pretreatment of EBV DNA plasma had larger tumor sizes. Ma et al. have already shown that EBV DNA was significantly correlated with tumor volume and volume of regional nodes (12). The close association between EBV DNA and tumor burden indicated the stage groups incorporating pretreatment with EBV DNA plasma could evaluate NPC more comprehensively than the other biomarkers (16).

In this study, T1WI_*Skewness*_ and T2WI_*Kurtosis*_ were significantly higher in the EBV DNA-negative group compared with those in the EBV DNA-positive group. Previous studies revealed Kurtosis derived from ADC maps correlated with p53 expression of the squamous cells in the head-and-neck of cases with carcinoma (HNSCC), and lower T2WI_*p*10_ or T2WI_*p*25_ correlated with hypoxia-inducible factor (HIF)-1α overexpression (17, 18). Bhatnagar et al. (19) suggested that a significant association existed between CE-T1WI_*Skewness*_ and microvessel density (MVD). Also, T2WI_*Kurtosis*_ was positively correlated with total cell counts (18); thus, the lower T2WI_*Kurtosis*_ in the EBV DNA-positive group of our study could be related to NPC tumor cell necrosis caused by EBV infection (20). Another study revealed that EBV infection promoted the chemokine (C-C motif) ligand 5 production, by increasing the expression of vascular endothelial growth factor (VEGF) and NPC angiogenesis by interacting with HIF-1α pathways (21). Moreover, greater MVD were observed in p53-positive tumors compared with those observed in p53-negative tumors (22); it has been proposed that p53 antagonizes HIF which induces hypoxia (23). Therefore, T1WI_*Skewness*_ and T2WI_*Kurtosis*_ were able to detect tumor heterogeneity, and higher T1WI_*Skewness*_ and T2WI_*Kurtosis*_ were potential indicators of hypoxia suppression, which is known to be associated with resistance to chemotherapy and RT, and poorer survival outcome (18).

Other studies have demonstrated that plasma EBV DNA has essentially become a tumor marker to predict prognoses and responses to various therapies in patients with NPC. Higher EBV DNA levels have been associated with poorer survival outcome (24, 25) and fewer distant metastases (3). In patients with NPC with detectable post-RT plasma EBV DNA, adjuvant chemotherapy did not improve relapse-free survival (RFS) (11, 26). On the contrary, post-RT plasma EBV DNA was associated with worse clinical outcomes, distant failure, and overall survival (OS) (13). In this study, MRI histogram and texture features were able to differentiate the EBV DNA-positive from the EBV DNA-negative NPC tumor groups and, thus, serve as *in vivo* and non-invasive imaging biomarkers, which can provide prognosis information for patients with NPC.

Texture analysis could quantitatively measure the intratumoral heterogeneity to discriminate tumor grades (27, 28). Several histogram and texture features showed significant differences when tumors with different T stages were compared, especially the distinct imaging features correlating with tumor heterogeneity, including T1WI_*Entropy*_, T1WI_*Contrastnt*_, and T2WI_*DiffVariance*_. No features derived from CE-T1WI showed statistical differences among the three groups; however, CE-T1WI_*DiffVariance*_ and CE-T1WI_5%_ were significantly higher in the T2 group compared with the T4 group. This result may be caused by the exclusion of the patients with T1 stage tumors due to difficulties in the segmentation of the small tumors. Therefore, it is absolutely essential to detect small tumors in early stages. Recent studies have shown that deep learning can assist in tumor staging and segmentation (29, 30). Ke et al. (31) revealed that the self-constrained 3D DenseNet model showed an ability to distinguish NPC tumors at any T stage from benign hyperplasia, with high overall accuracy of 97.77%. Artificial intelligence (AI) tools could improve the accuracy of NPC staging and segmentation; therefore, we will collect more data with small tumor for future studies.

We demonstrated the combination of overall stage, tumor volume, T2WI_*Kurtosis*_, and EBV status was the most effective model to predict PD in patients with NPC. Existing studies have used a few principal biomarkers as predictive tools for personalized therapy and survival status in patients with NPC, including EBV DNA level and imaging features. Radiomics nomogram combined radiomic features (8 CE-T1WI and 7 T2WI features) and clinical variables to provide pretreatment evaluations of local recurrences in patients with NPC (32). In patients with stage I–II NPC, pretherapy plasma EBV DNA level >4000 copies/ml was considered as poor risk indicators with a probability that distant failures would occur (33). Mao et al. (10) proved that higher CE-T1WI-based uniformity was an independent predictor of PFS and those patients with NPC could be clustered into four distinct survival group patterns based on multi-modality MRI radiomics (9). Pretreatment EBV DNA levels associated with 3-year PFS and overall survival (OS) in patients with NPC. The survival period was significantly higher in patients who achieved an undetectable EBV DNA level after treatment compared with patients with detectable EBV DNA (11). Moreover, radiomic signatures of pretreatment morphologic MRI could predict early responses to chemotherapy inductions in patients with NPC (34). However, limited studies have combined the histogram and texture features, and EBV DNA, to comprehensively evaluate NPC in clinical practice. In this study, not only EBV DNA status but also MRI histogram and texture imaging features were included in the predictive model for the prognosis of patients with NPC, according to previous studies mentioned above. To our knowledge, less previous studies combined EBV DNA status and imaging features to predict prognosis in patients with NPC. We think this clinical model could provide more predictive information for the clinicians.

This study has several limitations. First, the sample size was relatively small, especially for the PD cases, and patients were enrolled from a single center. The small sample size leads to no validation cohort in this study. The data imbalance can also cause inaccuracy of the statistical results. So the reliability and reproducibility of the predictive model should be validated by larger sample sizes from multiple centers. Second, only pretreatment EBV DNA samples were collected in this study. However, the dynamic changes in EBV DNA levels were also clinically significant as the decay of plasma EBV DNA during the latter part of therapy regimens could reflect a decrease in the number of tumor cells (35). More comprehensive information of EBV DNA should be collected, and maybe the changes in EBV DNA levels will influence the predictive model. Third, the follow-up period was relatively short, and the median PFS was only 23 months. With longer follow-up time, we can use survival analysis for more reliable predictive models. Fourth, we extracted only the seven first-order histograms and four texture-based features; more advanced radiomics and machine learning methods will be applied in further studies to obtain superior results and improve the reliability of the predictive model.

In conclusion, our study showed that larger tumor volume, T1WI_*Skewness*_, and T2WI_*Kurtosis*_ were associated with EBV DNA-negative status. Based on these findings, we developed a simple predictive model integrated MRI features and EBV status to predict and evaluate PD in patients with NPC. The predictive model can be used as a non-invasive and cost-effective detection method, and it also served as a visual tool to identify high-risk individuals with PD who would benefit from aggressive therapeutic strategies.

## Data Availability Statement

The raw data supporting the conclusions of this article will be made available by the authors, without undue reservation.

## Ethics Statement

The studies involving human participants were reviewed and approved by Fudan University Shanghai Cancer Center. The ethics committee waived the requirement of written informed consent for participation.

## Author Contributions

YG carried out the concepts and design of the study. QiaoL and TW carried out statistical analysis, literature research, manuscript editing and contributed equally to this work. PL provided information about the patients. YH, QinL, and YZ provided assistance for data acquisition. CF and RG provided the permission for imaging acquisition. All authors have reviewed the final version of the manuscript and approved it for publication.

## Conflict of Interest

RG was employed by Siemens Healthcare. CF was employed by Siemens Shenzhen Magnetic Resonance Ltd. The remaining authors declare that the research was conducted in the absence of any commercial or financial relationships that could be construed as a potential conflict of interest.
