# Supplementary material for: Whole-Tumor Histogram and Texture Imaging Features on Magnetic Resonance Imaging Combined With Epstein-Barr Virus Status to Predict Disease Progression in Patients With Nasopharyngeal Carcinoma
Source: Front Oncol. 2021 Mar 9;11:610804. doi: 10.3389/fonc.2021.610804 (PMC7986723; doi:10.3389/fonc.2021.610804)
Supplement: Supplementary file 1 [file Table_1.docx]

| SUPPLEMENTARY TABLE 1. A comparison of histogram and texture features within the different T stage groups | | | | | | | | |
| --- | --- | --- | --- | --- | --- | --- | --- | --- |
| Variable | T stage | | |  | P value | | | |
|  | T2 | T3 | T4 |  | P1 | P2 | P3 | P |
| Tumor volume (cm^3^) | 5.4  (4.1-7.4) | 8.1  (4.5-12.0) | 14.7  (10.6-21.1) |  | 0.216 | **0.000** | **0.008** | **<0.0001** |
| T1WI-based parameters |  |  |  |  |  |  |  |  |
| Mean | 302.1  (280.4-354.2) | 273.2  (257.7-293.5) | 266.3  (250.5-285.4) |  | **0.007** | **<0.0001** | 0.220 | **<0.0001** |
| SD | 69.0  (49.7-89.6) | 46.5  (38.1-56.5) | 48.4  (41.1-56.2) |  | **<0.0001** | **0.005** | 0.521 | **0.001** |
| Median | 294.5  (275.5-321.5) | 277.0  (255.0-295.8) | 264.5  (249.0-285.0) |  | **0.025** | **<0.0001** | 0.139 | **0.001** |
| 5% | 204.5  (183.5-236.5) | 207.5  (183.8-234.8) | 199.0  (180.0-216.8) |  | 1.000 | 0.179 | **0.070** | 0.305 |
| 95% | 419.5  (363.5-516.5) | 353.0  (328.5-381.3) | 339.0  (320.8-379.3) |  | **<0.0001** | **<0.0001** | 0.563 | **<0.0001** |
| Skewness*10^-2^ | 109.9  (42.5-182.8) | 53.3  (9.1-183.8) | 63.4  (33.1-172.1) |  | 1.000 | 1.000 | 1.000 | 0.407 |
| Kurtosis*10^-2^ | 451.0  (116.1-913.4) | 335.6  (245.1-778.0) | 358.2  (238.6-762.5) |  | 1.000 | 1.000 | 1.000 | 0.967 |
| Difference Entropy*10^-2^ | 81.7  (72.8-103.9) | 73.8  (67.0-87.6) | 73.2  (64.8-89.8) |  | **0.033** | 0.042 | 0.962 | 0.05 |
| Difference Variance*10^-2^ | 21.3  (16.4-31.5) | 18.3  (14.9-21.7) | 18.1  (13.6-24.3) |  | 0.329 | **0.027** | 0.894 | 0.059 |
| Contrast*10^-2^ | 52.0  (32.8-79.4) | 38.1  (29.5-47.8) | 38.5  (25.5-53.4) |  | **0.021** | **0.021** | 0.963 | **0.025** |
| Entropy*10^-2^ | 136.6  (111.4-163.9) | 116.6  (97.2-132.0) | 115.5  (83.0-142.4) |  | **0.022** | **0.019** | 0.915 | **0.024** |
| T2WI-based parameters |  |  |  |  |  |  |  |  |
| Mean | 534.1  (488.1-556.6) | 498.9  (464.4-526.7) | 460.0  (414.5-496.9) |  | 0.073 | **<0.0001** | 0.052 | **0.001** |
| SD | 104.9  (96.0-129.3) | 116.5  (95.3-134.3) | 122.6  (98.9-134.1) |  | 1.000 | 1.000 | 1.000 | 0.625 |
| Median | 535.5  (483.5-566.5) | 500.5  (462.8-537.5) | 451.5  (408.0-496.3) |  | **0.046** | **<0.0001** | **0.032** | **<0.0001** |
| 5% | 318.5  (273.5-374.5) | 298.5  (271.8-346.3) | 270.5  (233.3-316.3) |  | 0.873 | 0.059 | 0.606 | 0.051 |
| 95% | 694.5  (649.5-743.5) | 671.5  (630.0-722.5) | 651.0  (594.0-694.8) |  | 0.842 | 1.000 | 1.000 | 0.074 |
| Skewness*10^-2^ | 37.7  (-37.4-40.6) | 36.7  (-4.5-72.2) | 42.8  (13.1-105.7) |  | 0.079 | 0.073 | 0.244 | 0.072 |
| Kurtosis*10^-2^ | 123.8  (68.0-250.6) | 128.0  (58.9-273.0) | 140.8  (28.6-346.9) |  | 1.000 | 1.000 | 1.000 | 0.975 |
| Difference Entropy*10^-2^ | 122.4  (112.7-132.9) | 115.7  (103.6-126.3) | 117.4  (106.0-127.0) |  | 0.584 | 0.409 | 1.000 | 0.167 |
| Difference Variance*10^-2^ | 49.3(41.9-57.9) | 40.8(35.0-52.5) | 42.1(33.8-48.8) |  | 0.064 | **0.023** | 0.656 | **0.049** |
| Contrast*10^-2^ | 160.1  (121.8-174.0) | 127.9  (103.5-177.1) | 124.7  (91.2-168.3) |  | 0.823 | 0.808 | 1.000 | 0.213 |
| Entropy*10^-2^ | 212.3  (194.0-226.6) | 198.2  (176.3-216.8) | 203.8  (179.5-219.0) |  | 0.665 | 0.755 | 1.000 | 0.215 |
| CE-T1WI-based parameters |  |  |  |  |  |  |  |  |
| Mean | 584.6  (536.2-651.0) | 566.1  (520.0-631.2) | 553.9  (487.2-589.4) |  | 0.798 | 0.057 | 0.372 | 0.254 |
| SD | 103.3  (90.2-118.2) | 99.9  (84.7-121.1) | 106.5  (87.7-117.7) |  | 0.713 | 0.973 | 0.741 | 0.921 |
| Median | 590.5  (536.5-654) | 567.5  (521.8-635.5) | 561.5  (491.8-591.8) |  | 0.755 | 0.080 | 0.393 | 0.280 |
| 5% | 389.5  (347.5-465.5) | 397.5  (355.5-439.6) | 355.5  (298.3-410.8) |  | 0.962 | **0.032** | 0.058 | 0.068 |
| 95% | 749.5  (663.5-823.5) | 712.5  (664.0-825.8) | 715.5  (621.8-767.0) |  | 0.779 | 0.149 | 0.285 | 0.335 |
| Skewness*10^-2^ | -25.5  (-48.1-[-5.7]) | -22.1  (-59.7-0.6) | -28.1  (-43.1-[-11.2]) |  | 0.810 | 0.734 | 0.961 | 0.942 |
| Kurtosis*10^-2^ | 63.1  (24.3-120.9) | 98.1  (16.0-149.1) | 161.6  (147.0-171.8) |  | 0.718 | 0.741 | 0.923 | 0.919 |
| Difference Entropy*10^-2^ | 164.9  (149.9-179.2) | 161.6  (149.8-174.0) | 161.6  (147.0-171.8) |  | 0.501 | 0.293 | 0.705 | 0.558 |
| Difference Variance*10^-2^ | 114.0  (75.2-144.2) | 95.9  (74.3-111.1) | 86.2  (64.2-108.8) |  | 0.173 | **0.026** | 0.277 | 0.066 |
| Contrast*10^-2^ | 384.0  (255.6-545.9) | 363.5  (253.8-482.4) | 336.4  (230.8-379.3) |  | 0.695 | 0.096 | 0.193 | 0.218 |
| Entropy*10^-2^ | 269.7  (260.3-295.4) | 272.3  (254.7-290.0) | 278.2  (253.8 -289.0) |  | 0.659 | 0.587 | 0.907 | 0.846 |
| P1: difference in values between the T2 and T3 groups. P2: difference in values between the T2 and T4 groups. P3: difference in values between the T3 and T4 groups. P: difference in values among the 3 groups. | | | | | | | | |
